# Phosphorylation impact on Spleen Tyrosine kinase conformation by Surface Enhanced Raman Spectroscopy

**DOI:** 10.1038/srep39766

**Published:** 2017-01-05

**Authors:** Maximilien Cottat, Ryohei Yasukuni, Yo Homma, Nathalie Lidgi-Guigui, Nadine Varin-Blank, Marc Lamy de la Chapelle, Christine Le Roy

**Affiliations:** 1 Université Paris 13, Sorbonne Paris Cité, Laboratoire CSPBAT, CNRS (UMR 7244), 74 rue Marcel Cachin, F-93017 Bobigny, France; 2 Université Paris 13, Sorbonne Paris Cité, Laboratoire ASIH, 74 rue Marcel Cachin, F-93017 Bobigny, France; 3 INSERM U978, Bobigny, France

## Abstract

Spleen Tyrosine Kinase (Syk) plays a crucial role in immune cell signalling and its altered expression or activation are involved in several cancers. Syk activity relies on its phosphorylation status and its multiple phosphorylation sites predict several Syk conformations. In this report, we characterized Syk structural changes according to its phosphorylation/activation status by Surface Enhanced Raman Spectroscopy (SERS). Unphosphorylated/inactive and phosphorylated/active Syk forms were produced into two expression systems with different phosphorylation capability. Syk forms were then analysed by SERS that was carried out in liquid condition on a lithographically designed gold nanocylinders array. Our study demonstrated that SERS signatures of the two Syk forms were drastically distinct, indicating structural modifications related to their phosphorylation status. By comparison with the atomic structure of the unphosphorylated Syk, the SERS peak assignments of the phosphorylated Syk nearest gold nanostructures revealed a differential interaction with the gold surface. We finally described a model for Syk conformational variations according to its phosphorylation status. In conclusion, SERS is an efficient technical approach for studying *in vitro* protein conformational changes and might be a powerful tool to determine protein functions in tumour cells.

Spleen Tyrosine Kinase (Syk) is a cytoplasmic tyrosine/serine kinase that plays a crucial role as signal transducer in immune cells^1^,^2^. Its functions and regulation are widely studied in normal cells and alterations of its expression/activation are implicated in several forms of cancer, making Syk an attractive target to exploit for therapeutic purposes^3^,^4^. Syk contains two Src homology domains at the amino- and carboxy-termini (Nt- and Ct-SH2) and a kinase domain; these 3 domains are connected by an inter-SH2 linker (inter-domain A) and an inter SH2-kinase linker (inter-domain B)^5^. Upon immune stimulation, Syk is able to bind specific phosphorylated receptor motifs (pITAMs) through its tandem SH2 domains. Also, Syk is able to phosphorylate itself. Both of these events are linked to an increase of its kinase activity^6^,^7^,^8^. Biochemical and enzymatic studies propose that Syk adopts a low activity conformation in absence of binding to ITAM or phosphorylation. Syk switches to an active conformation in presence of either one or both stimuli^8^,^9^,^10^. X-ray crystallography of unphosphorylated Syk forms^8^,^11^,^12^ (isolated tandem SH2 domains^8^, kinase domain^11^ and full-length^12^) reinforces this model by proposing that unphosphorylated Syk adopts an auto-inhibited conformation, which is maintained by hydrogen bond interactions between both inter-domains as well as by interactions between the inter-domain A and the kinase domain^12^. Disruption of these inhibitory interactions upon pITAM binding or phosphorylation leads to the release from the auto-inhibited conformation to its kinase active conformation^12^, even when minor conformational changes occur as shown by low-resolution electron microscopy studies^13^,^14^. Activated Syk contains multi-phosphorylation sites, including phospho-tyrosines 323, 348/352 and 525/526^12^,^15^,^16^,^17^. Despite the lack of crystallographic analysis of phosphorylated/activated Syk, the presence of multiple phosphorylated sites and the various biological activities predict that the kinase might adopt more than 2 conformations, *i.e.* the unphosphorylated and phosphorylated forms.

To have insights into such protein conformational variations, the Surface Enhanced Raman Spectroscopy (SERS) based technique is an effective approach. In SERS, Raman scattering efficiency is drastically enhanced at the surface of noble metal nanostructures by the generation of a locally enhanced electromagnetic (EM) field following excitation of Localized Surface Plasmon Resonance (LSPR)^18^. The resulting SERS vibration spectrum represents the physicochemical state of the molecule with single molecular level sensitivity under optical microscope^19^,^20^,^21^,^22^. These SERS properties permit signal acquisitions from a small volume of dilute protein solutions^23^,^24^,^25^,^26^,^27^ and, thus offer the advantage of studying various protein statuses compared to other methods for protein structural analysis.

In this work, we applied the SERS based technique to analyse Syk conformational changes related to its phosphorylation status. After evaluating both phosphorylation and kinase activity levels of Syk produced in two distinct expression systems, the SERS spectra were acquired in liquid on a gold nanocylinders array. SERS signatures of phosphorylated and unphosphorylated Syk forms, which reflected interaction between gold surface and Syk amino acid residues exposed at the surface of the nanostructure, were drastically different. With the help of atomic structural data of unphosphorylated Syk, SERS results allowed us to propose a model of Syk conformational changes according to its phosphorylation/activation.

## Results

### Syk phosphorylation level is linked to its kinase activity *in vitro*

To study Syk structural variations as a function of its phosphorylation status, we first confirmed the relationship between the phosphorylation status and the kinase activity with a wild type (WT) human Syk protein produced either in prokaryotic BL21 bacteria or in eukaryotic Sf9 insect cells systems. Syk expression and its global tyrosine phosphorylation levels were detected by western blot using anti-Syk (α-Syk) and anti-phosphotyrosine antibodies (α-pY), respectively.

**Figure 1:**
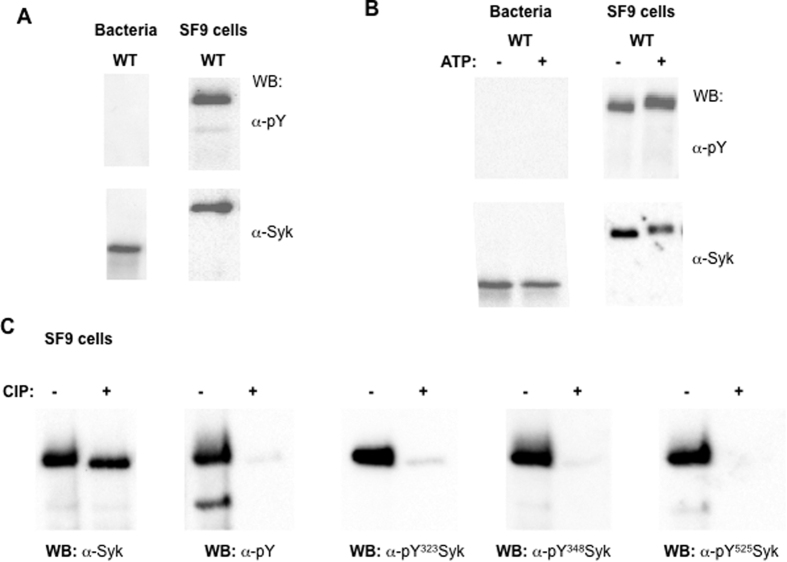
Phosphorylation and activation levels of Syk produced in bacteria and insect cells. Constructs encoding human wild type (WT) Syk were expressed in BL21 bacteria (**A** and **B**) and Sf9 insect cells (**A**,**B** and **C**). Kinase assay in presence (+) or not (−) of Adenosine Triphosphate (ATP) was used to measure Syk self kinase activity that was reflected by tyrosine phosphorylated (pY)-Syk (**A** and **B**). Phosphatase treatment with calf intestinal alkaline phosphatase (CIP) was used to transform P-Syk into deP-Syk (**C**). Produced Syk forms were separated on SDS-PAGE gel and Syk expression and phosphorylation were revealed with the indicated antibodies anti-Syk (α-Syk), anti-phospho-tyrosine antibody (α-pY) and anti-phospho-specific antibodies (α-pY-^323^, -^348^ and -^525^).

Due to the lack of post-translational modifications machinery in bacteria^28^, Syk expressed in BL21 did not show any phosphorylation signal whereas Syk expressed in Sf9 cells was phosphorylated (Fig. 1A). Of note, the apparent variation of molecular weight between the proteins produced in BL21 and Sf9 was attributed to the different tags (His and Glutathione S-Transferase (GST)-His, respectively). In presence of Adenosine Triphosphate (ATP), auto-phosphorylation activity of both forms was evaluated; no phosphorylation was detected for Syk protein produced in BL21 bacteria while an enhanced phosphorylation of Syk was observed when produced in Sf9 cells (Fig. 1B). Thus, our results demonstrated that the initial phosphorylation of Syk allowed a kinase activity that was corroborated by the detection of specific phospho-tyrosine residues, such as Y323, Y348 and Y525, in the Syk produced in Sf9 cells (Fig. 1C). Treatment with the calf intestinal alkaline phosphatase (CIP) caused a severe decrease of the global and specific phospho-tyrosine levels (Fig. 1C), thus generating a dephosphorylated form of Syk isolated from Sf9 cells (called deP-Syk).

Altogether, these data demonstrated that Syk protein produced in bacteria was un-phosphorylated and inactive (called unP-Syk) whereas that produced in insect cells was phosphorylated and kinase active (called P-Syk).

### SERS spectra of Syk depend on its phosphorylation and kinase activity status

After having checked the quality of the fabricated nanostructures (Fig. S1), SERS experiments were carried out in liquid condition on a gold nanocylinders array in order to obtain structural information on the above-described Syk forms. The contribution of the additional GST tag to the overall Syk spectral signature was checked by comparing SERS spectra of the deP-Syk (GST-His P-Syk produced in Sf9 cells and then treated with the CIP) and unP-Syk (His-Syk produced in BL21 bacteria). Moreover, a kinase-inactive mutant of Syk (indicated as KD-Syk), which exhibited a similar sequence to the WT-Syk except for mutated amino acids in the kinase domain that interfere with Syk ability to bind to ATP^29^, was also exploited. As expected, the KD-Syk form produced in BL21 bacteria was not phosphorylated in presence or absence of ATP (data not shown) and was used as reference for structural modifications.

**Figure 2:**
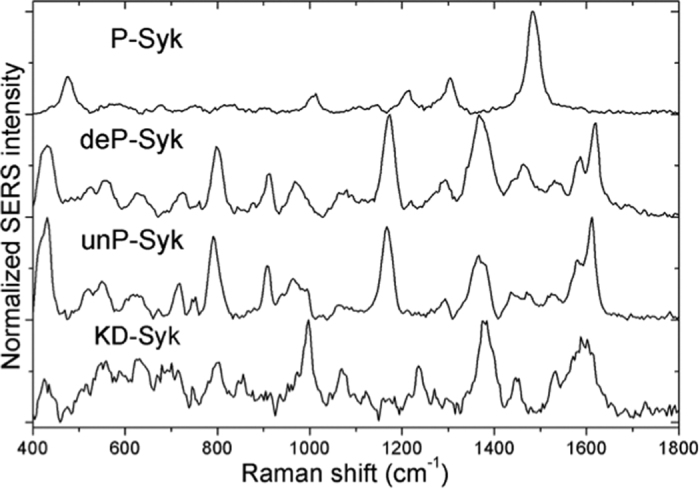
SERS spectra of KD-, unP-, deP- and P-Syk forms. For each Syk form, spectra were acquired at five locations on the same array. After correcting background, measures obtained the spectra were averaged and normalized at their maximum. The baseline of each spectrum was offset for clarity.

The SERS spectra of the four Syk samples, whose biophysical properties are recapitulated in Table 1, are depicted in Fig. 2. Global comparison of four spectra revealed similar peaks below 800 and over 1300 cm^−1^ for unP-, deP- and KD-Syk whereas P-Syk had a radically different SERS spectrum. With similar biochemical properties in terms of phosphorylation and kinase activity statuses, unP-Syk and deP-Syk samples showed rather equivalent spectral features. Importantly, this common spectral signature strongly excluded a contribution of the expression system used (bacteria *vs* insect cells) as well as the tags (His *vs* GST-His). Indeed, SERS spectrum of the GST protein (Fig. S2) argued for a very mild influence of the GST tag in the overall spectral signature of the P-Syk. Moreover, spectrum reproducibility for each sample indicated a specific interaction of each Syk form with the gold surface.

The main peaks of the four Syk spectra were assigned (listed in the Table S1) with a particular interest for those corresponding to sulfurs, aromatic amino residues and polypeptide backbones, usually characterized in protein Raman spectra^20^,^21^,^25^,^27^,^30^,^31^.

In the spectral region below 750 cm^−1^, sulfur related vibrations showed characteristic peaks. Peaks around 420 cm^−1^ and from 510 to 560 cm^−1^ were assigned to the disulfide S-S stretching vibration, linked to two cysteine (Cys) residues nearby. Peaks around 620 and 715 cm^−1^ correspond to C-S stretching vibration. All these peaks were detected in the spectra of KD-, unP- and deP-Syk but not in the spectrum of P-Syk. According to the recent atomic structural analysis of unP-Syk^12^, which contains nine Cys residues in its amino acid sequence, only one disulfide bond might exist between the Cys593 and Cys597 that are spatially close enough in the kinase domain (Fig. S3). Thus, the detection of the intramolecular disulfide bond in the KD-Syk, unP-Syk and deP-Syk SERS spectra is in favour of an adsorption of Syk to the gold surface through its kinase domain. Further comparison of the SERS spectra with the data provided by the Syk atomic structure^12^ revealed that another Cys (Cys101), which is located at the N-terminal end of the Syk interdomain A, was present in the plane containing the disulfide bond (Fig. S3). Indeed, the peak detected at 230 cm^−1^ on the Rayleigh scattering background in the unphosphorylated Syk spectra could be assigned to S-Au vibration by a contact of the thiol group of the Cys101 to the gold surface (Fig. S4). To quantify the affinity strength of the disulfide bond and the thiol group to the gold surface, the accessibility and surface energy of sulfur atoms of Cys101, 593 and 597 were estimated based on the solvent molecular surface calculations (Fig. S5). Values for the targeted Cys residues demonstrated their accessibility to the solvent. Their low surface energy suggested that the link between sulphur atoms of Cys residues and gold surface was not the main driving force in the Syk-gold nanostructure interactions and might play a role in the Syk orientation once it was adsorbed on the surface.

The bands associated with amino acids carrying aromatic rings in their side chains, such as tryptophan (Trp), tyrosine (Tyr), phenylalanine (Phe) and histidine (His), are usually the main components of protein SERS spectra. Peaks around 750, 1530, 1580 cm^−1^ for Trp, 645 and 800 cm^−1^ for Tyr, 995 cm^−1^ for Phe, as well as the strong contribution of mixed ring stretching modes around 1615 cm^−1^, were commonly found in KD-, unP- and deP-Syk. Peaks at 845 and 1236 cm^−1^, which are assigned to Tyr, were present in the KD-Syk only. The latter contains a single mutation of the Syk kinase domain (Lys402 to Ala) that renders the kinase inactive^29^. Comparison of KD-Syk and unP-Syk SERS spectra revealed differences in the range between 800 and 1300 cm^−1^ that included peaks assigned to aromatic amino acid residues that were not substituted by the mutation. Therefore, the observed spectral distinctions, which probably result from structural modifications caused by the mutation, implied proximity of the Syk kinase domain to gold nanostructures. This model was reinforced by the presence of peaks corresponding to a disulfide bond between two Cys residues in KD-Syk, unP-Syk and deP-Syk spectra as described above. While peaks around 965 cm^−1^ for Trp and 1170 cm^−1^ for Tyr or Phe were detected in both unP- and deP-Syk, small contributions from Phe or Trp were detected at 1014 cm^−1^ in the P-Syk spectrum.

To further analyse our Syk SERS spectra, we next focused our attention on the peaks from aliphatic carbons that were also observed between 1290 and 1490 cm^−1^ and were assigned to aliphatic amino acids with bulky hydrocarbons, such as valine (Val), leucine (Leu) and isoleucine (Ile). These spectral features were strongly detected in all Syk spectra and dominant for the P-Syk spectrum. In contrast, the amide I bands around 1630–1680 cm^−1^, which are typical of protein secondary structure originated from polypeptide backbones, were poorly or not detected in all Syk spectra. The signal from amide I bands are normally the strongest among other amide bands, therefore bands around 1550 cm^−1^ for amide II and from 1230–1300 cm^−1^ for amide III should have little contribution to all the spectra. Thus, these silent amide bands were likely to come from the large distance of the Syk peptide bonds from the gold surface^32^. Based on the analysis of the Syk atomic structure^12^, the disulfide bond between the Cys593 and Cys597 residues and the Cys101 residue were surrounded by aromatic amino acids (Tyr, Trp) and aliphatic amino acids with bulky side chains (Leu, Val) (Fig. S6); their signals were effectively confirmed in the Syk SERS spectra. Acting as spacers between the peptide bonds and gold surface, the long length of these amino acid side chains might result in the suppression of amide signal in the SERS spectra of Syk.

Collectively, global and detailed comparisons of the four Syk SERS spectra showed differential patterns corresponding to the structural variations of their biophysical properties especially between unphosphorylated/inactive Syk and phosphorylated/active Syk.

## Discussion

SERS spectrum mainly reflects adsorbed part of proteins onto gold nanostructures with an EM field generated by LSPR excitation exponentially decaying from a gold surface^33^. Based on this SERS advantage, we discussed interactions between the various Syk forms with gold nanostructures in order to establish a model that links structural changes and phosphorylation status of the protein.

The phosphorylation impact on Syk structure was then examined based on the distinct SERS spectra between unP-Syk and P-Syk. Interestingly, these spectral variations were larger than the ones observed between unP-Syk and KD-Syk, indicating that Syk phosphorylation allowed a larger structural change as compared to those created by a point mutation in the Syk kinase domain. The structural change resulted in another interaction between P-Syk and the gold surface. Indeed, peaks corresponding to thiol and disulfide bonds were no longer detected in P-Syk, reflecting a large modulation of the link between Syk and the gold surface. Moreover, peaks from the Tyr and Trp residues were weakly detected in P-Syk spectrum, revealing that these amino acids were not anymore in close proximity to the gold surface. Furthermore, aliphatic carbon signatures dominated the P-Syk SERS spectrum and their hydrophobic properties might explain how Syk interacts with the gold surface. Thus, the hydrophobic part that is folded inside and masked in the compact structure of Syk might be uncovered following phosphorylation. Flexible interdomains are thought to induce a local conformational change through phosphorylation of tyrosine residues in these regions^17^. This model is supported by the presence of fewer aromatic amino residues in the interdomains as seen in its primary structure.

**Figure 3:**
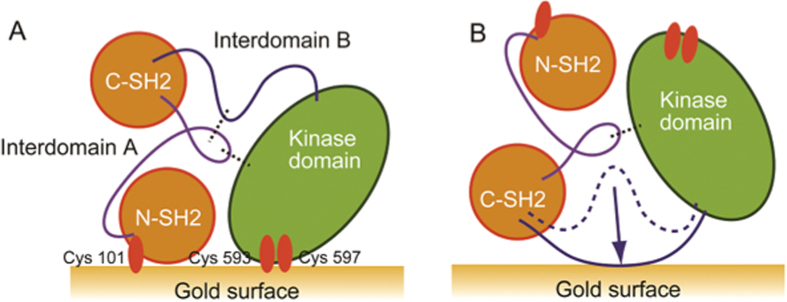
Model of phosphorylation-dependent Syk conformational changes based on SERS spectra analysis. This model shows SH2 domains in orange, the interdomain A in purple, interdomain B in blue and kinase domain in green. Dotted lines show hydrogen bonds between interdomains as well as between the interdomain A and kinase domain. (**A**) Auto-inhibited form of unphosphorylated Syk adsorbed onto gold surface through Cys101 in its N-SH2 domain and through Cys593-Cys597 disulfide bond in its kinase domain. (**B**) Active Syk form of phosphorylated Syk adsorbed onto gold surface through hydrophobic interactions after motion of its interdomain B.

Our results are summarized and illustrated in Fig. 3. Our model of phosphorylation-dependent conformational changes shows the unphosphorylated and phosphorylated forms of Syk, and how they interact with the surface of the gold nanostructures. A Syk crystallographic study^12^ and the previous Syk activation model^7^ proposed that unphosphorylated Syk exhibits a closed conformation with both interdomains folded inside the protein; this structure is stabilized by hydrogen bonds between both interdomains, as well as between the interdomain A and the kinase domain. Our spectral data revealed that unphosphorylated Syk interacts with the gold surface through Cys101 at the N-terminal end of the interdomain A and through a disulfide bond (Cys593-Cys597) in the kinase domain (Fig. 3A). The observed SERS spectral changes after phosphorylation suggest that the enhanced hydrophobic properties of phosphorylated Syk play a role in its interaction to the gold surface as a result of an inside-out movement of the phosphorylated interdomains (Fig. 3B). A previous study on the phosphorylated structure of Syk by low resolution single particle electron microscopy, concluded to a modest displacement between both SH2 and kinase domains with no disruption of the hydrogen bond between interdomain A and kinase domain^13^,^14^. Our results rather argued for a larger rearrangement of the hydrophobic region of the interdomain B to release Syk from its inhibited closed conformation. Supporting this hypothesis, a similar conformational change has been proposed for the activation of Zap70 kinase^34^, the second member of the Syk kinase family, that presents high homology both in the structure and the activation process with Syk (Fig. 3B).

In this report, we studied the conformational differences between phosphorylated and unphosphorylated forms of the protein. It is worth noting that spectral similarities between unP-Syk and deP-Syk suggested that phosphorylated Syk was able to reverse its conformation once it was unphosphorylated following phosphatase treatment. Additionally, the presence of multiple phosphorylation sites in Syk suggested intermediate phosphorylation statuses that might translate into diverse SERS spectra. In a preliminary study, SERS spectra of other Syk forms, which showed intermediate phosphorylation levels between unP-Syk and P-Syk species by western blot (data not shown), shared spectral features of both unP-Syk and P-Syk. Indeed, a gradual transition of the SERS peaks from unP-Syk to P-Syk was observed when the phosphorylation levels were increased (Fig. S7). Without ruling out the possibility that these SERS spectra were originated from mixed spectra of unP-Syk and P-Syk in different ratios, the emergence of new peaks (absent in both unP-Syk and P-Syk spectra) suggested the detection of Syk species with intermediate phosphorylation status. This funding is in agreement with a quantitative high-resolution mass spectrometry study that revealed different phosphorylation kinetics upon immune stimulation of multiple Syk phosphorylation sites. While the specific phospho-tyrosine residues 323, 348 and 525 are grouped as early phosphorylation sites, other amino acid residues are grouped as late phosphorylation sites and contribute to regulate the diverse biological functions of Syk^16^. Thus, future analysis of a series of Syk conformational modifications, associated with its specific phospho-residue patterns, should reveal the dynamic properties of Syk structures along its activation and its biological functions in normal and pathological contexts.

## Methods

### Protein expression

His-tag human unphosphorylated wild type (WT) (unP-Syk) and kinase-dead (KD-Syk) Syk recombinant proteins were produced in BL21 bacteria (Invitrogen) and purified over magnetic Nickel beads (Millipore). GST-His WT phosphorylated Syk form (P-Syk) was produced in baculovirus infected Sf9 cells and purified (Active Motif). Purified proteins were then concentrated using columns with a 30 kDa-cut-off (Millipore) before loading by SDS-PAGE for western blot analysis and onto gold nanostructures for SERS analysis.

### Kinase assay

UnP-Syk and P-Syk were incubated in a buffer containing 50 mM Tris-HCl, 25 mM MgCl_2_, 10 mM NaVO_3_, 1 mM DTT, supplemented or not with 100 mM ATP (Sigma). After 1 hour incubation at room temperature, reactions were stopped by cooling down on ice. Unphosphorylated and phosphorylated Syk forms were separated by SDS-PAGE, transferred into nitrocellulose membrane and blots incubated with monoclonal mouse anti-phosphotyrosine antibody (α-pY; Millipore), mouse anti-Syk antibody (α-Syk; Becton Dickinson) or rabbit anti-phospho-specific tyrosine Syk antibodies (α-pY^323^ Syk, α-pY^348^ Syk, α-pY^525^ Syk; Cell Signalling) followed by a goat anti-mouse or -rabbit secondary antibodies coupled to Horse Radish Peroxidase enzyme (BioRad) before revelation with the Enhanced Chemiluminescence kit (Pierce).

### Phosphatase assay

P-Syk was incubated in the presence or not of 10 units of Calf Intestinal Phosphatase (CIP) in a buffer containing 50 mM Tris-HCl and processed as described in the kinase assay (*cf*. above).

### SERS experiment

A gold nanocylinder array (diameter 130 nm, height 50 nm, gap 200 nm) was built as SERS substrates by electron-beam lithography following a classical lift-off process on a glass substrate with 3 nm of chromium adhesion layer. Ten microliters of protein solution were deposited on the gold nanocylinder substrate and incubated for 18 hours at 4 °C in a humidified chamber. The substrate was set nanostructured side down on a glass substrate with a cavity filled with PBS 1X. SERS spectra were recorded with a commercial Raman microspectrometer (Horiba Jobin-Yvon, Xplora). The excitation laser (λ = 660 nm) was focused through a microscope 60× objective (Olympus, NA: 0.6) and Raman scattering was collected through the same objective. The laser power was 1 mW at the sample position. For one SERS spectrum, signal was accumulated twice for 60 sec.

## Additional Information

**How to cite this article**: Cottat, M. *et al*. Phosphorylation impact on Spleen Tyrosine Kinase conformation by Surface Enhanced Raman Spectroscopy. *Sci. Rep.* **7**, 39766; doi: 10.1038/srep39766 (2017).

**Publisher's note:** Springer Nature remains neutral with regard to jurisdictional claims in published maps and institutional affiliations.

## Supplementary Material

Supporting Information

## Figures and Tables

**Table 1:** Biophysical properties of the Syk samples.

Sample	Tag	Expression system	Phosphorylation status	Kinase activity
P-Syk	GST-HIS	Sf9 cells	phosphorylated	active
deP-Syk	GST-HIS	Sf9 cells	unphosphorylated	inactive
unP-Syk	HIS	BL21 bacteria	unphosphorylated	inactive
KD-Syk	HIS	BL21 bacteria	unphosphorylated	dead

For the four Syk samples used in this study (first column), which were differentially tagged (second column) and produced in insect cells or bacteria (third column), their phosphorylation status (fourth column) and self-kinase activity (fifth column) are indicated based on the results shown in Fig. 1.
