# Supplementary material for: Phosphorylation impact on Spleen Tyrosine kinase conformation by Surface Enhanced Raman Spectroscopy
Source: Sci Rep. 2017 Jan 5;7:39766. doi: 10.1038/srep39766 (PMC5214100; doi:10.1038/srep39766)
Supplement: Supporting Information [file srep39766-s1.pdf]

Supporting information

Phosphorylation impact on Spleen Tyrosine Kinase conformation by Surface Enhanced Raman Spectroscopy

Maximilien Cottat[1,2,3], Ryohei Yasukuni[1], Yo Homma[1,2,3], Nathalie Lidgi-Guigui[1], Nadine Varin-Blank[2,3], Marc Lamy de la Chapelle[1,*] and Christine Le Roy[2,3,*]

[1]Université Paris 13, Sorbonne Paris Cité, Laboratoire CSPBAT, CNRS (UMR 7244), 74 rue Marcel Cachin, F-93017 Bobigny, France

[2]Université Paris 13, Sorbonne Paris Cité, Laboratoire ASIH, 74 rue Marcel Cachin, F-93017 Bobigny, France

[3]INSERM U978, Bobigny, France

*Co-corresponding authors: marc.lamydelachapelle@univ-paris13.fr and christine.le-roy@inserm.fr

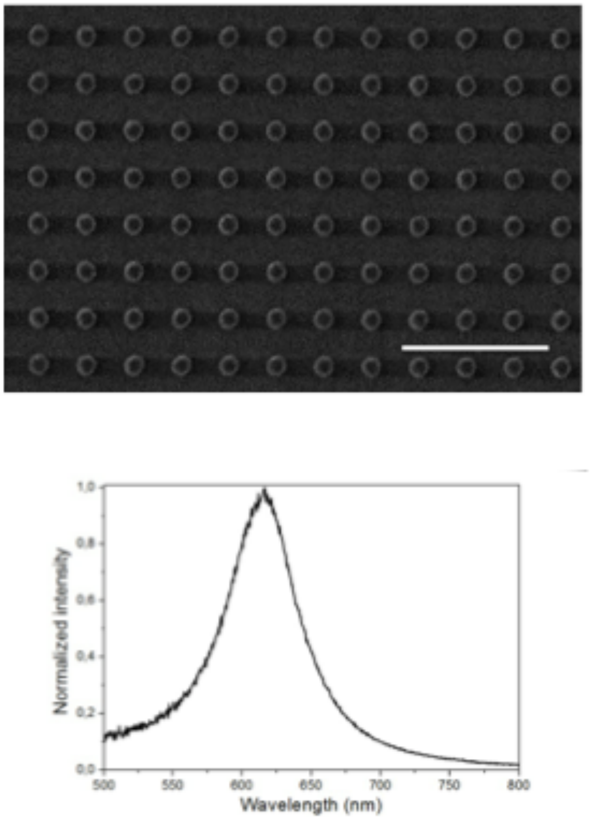

Figure S1. SEM image and extinction spectrum of the gold nanocylinders. Top, scanning electron microscopy (Zeiss SEM) of the Au nanostructures (diameter 130 nm, height 50 nm, gap 200 nm in both directions) that were built as SERS substrates by electron-beam lithography following a classical lift-off process on a glass substrate with 3 nm of chromium adhesion layer. Bar scale equal 1 μm. Bottom, the extinction spectrum for the gold nanocylinders is plotted and exhibits a localized surface plasmon resonance (LSPR) at 620 nm as expected for such nanostructures. The extinction spectrum was recorded in dry condition. As the SERS experiments were performed in liquid environment, the LSPR is shifted of several tens of nanometers to match the SERS excitation wavelength at 660 nm.

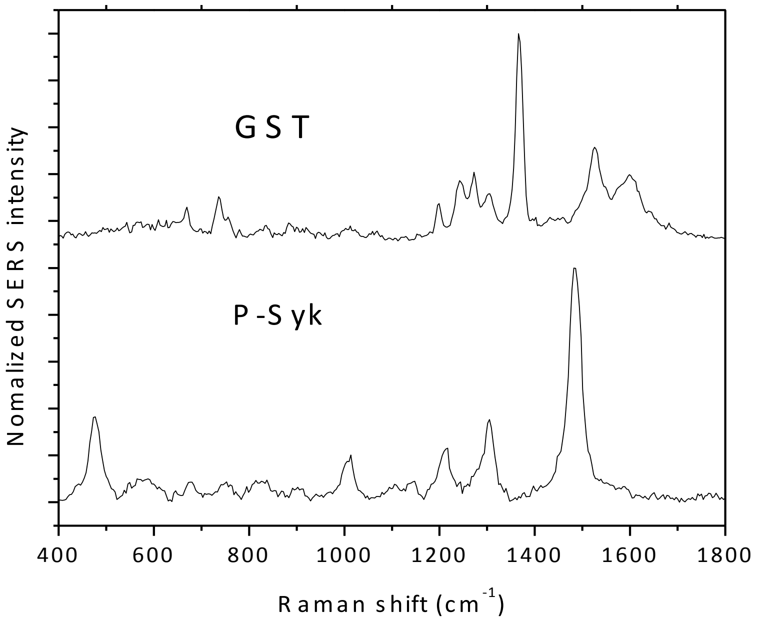

Figure S2. Comparison between normalized SERS spectra of the GST and GST-His coupled P-Syk proteins. Ten μL of 0.1 mg/mL GST protein solution (Sigma Aldrich) were deposited on a SERS substrate and incubated for 1 hour at room temperature. The excitation laser (λ = 660 nm, 0.4 mW) was focused through a 100 x objective microscope. SERS spectrum of the GST was acquired twice for 60 seconds. The more intense peak of GST (at 1366 cm^{-1}) was not detected in the SERS spectrum of P-Syk, indicating very low contribution of the GST in the P-Syk SERS spectrum.

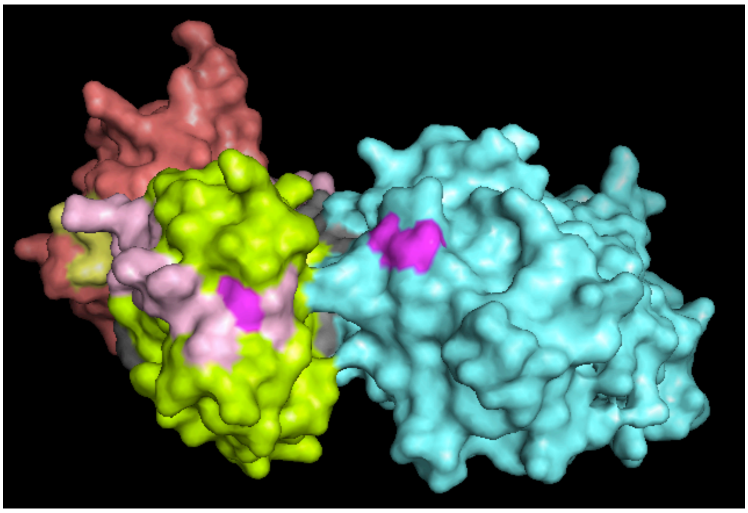

Figure S3. Exposed Cys residues (dark pink) at the surface of the Syk structure. Unphosphorylated Syk structure (PDB 4FL2 Syk[1]) showed the Cys101 in the Nt-SH2 domain (lemon), as well as Cys593 and Cys597 in the kinase domain (light blue), in the same plane. This model was created by the MacPyMol software.

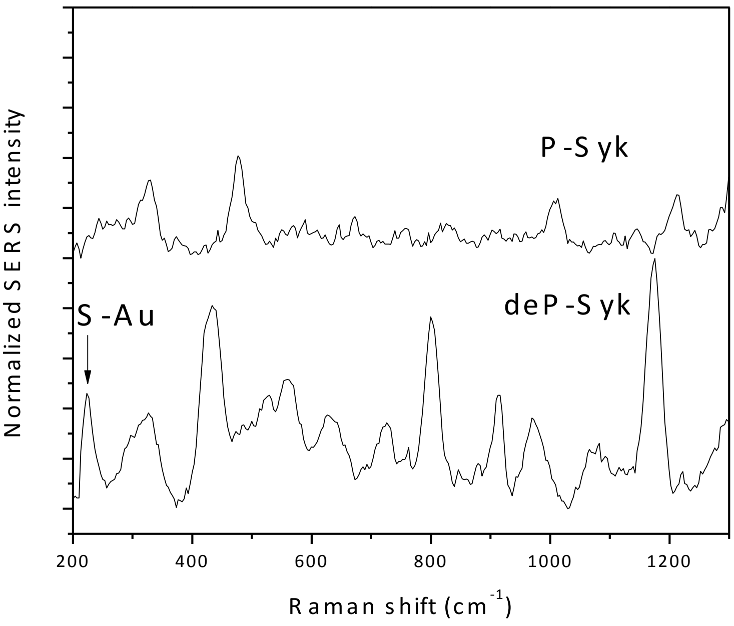

Figure S4. Normalized SERS spectra of deP-Syk and P-Syk in a low frequency spectral region. The Sulfur-Gold vibration mode (S-Au as indicated with an arrow) was observed at around 230 cm^{-1} in the deP-Syk spectrum only.

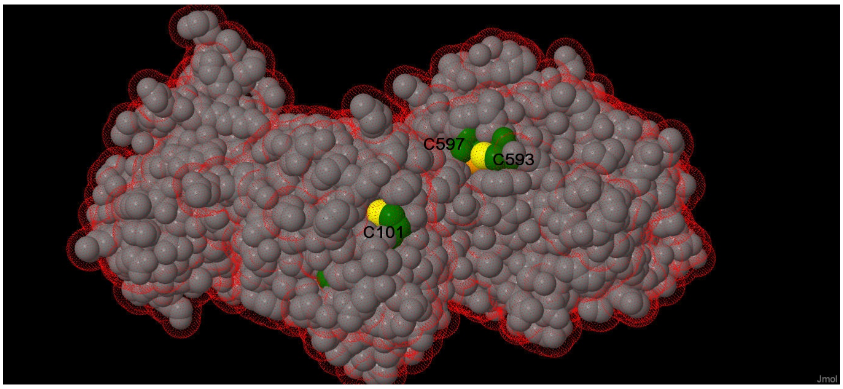

Figure S5. CPK view of the Syk protein. Based on the PDB 4FL2 Syk[1], the three cysteine residues and their sulfur atoms are coloured in green and in yellow, respectively (the sulfur atom in Cys597 was coloured in dark yellow-orange to be distinguished). The Solvent Molecular Surface Area (MSA)[2] of Syk, materialized by the red dots and defined as the contact surface area of a solvent probe sphere that rolls along the surface of the protein, was obtained using Jmol[3] with a solvent probe of 1.2 Å. Over the three Cys residues, two accessible sulphurs of the Cys101 and Cys593 were graphically confirmed. A surface energy calculation using GetArea[4] showed that only the sulphur of Cys593 has enough surface energy to bind to a gold nanoparticle in the crystalline state. The sulphur of Cys101 may bind to a gold surface through a fluctuated motion in a solvent.

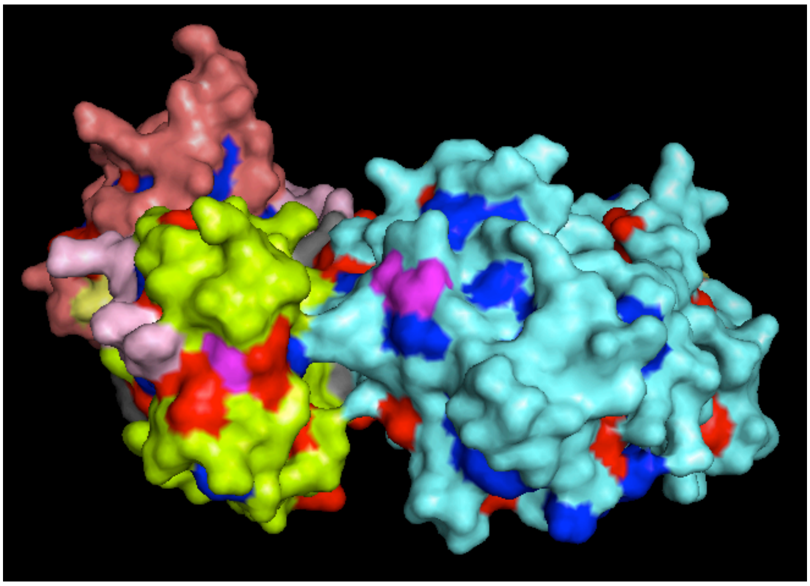

Figure S6. Visualization of specific amino acid residues surrounding the three exposed Cys residues at the surface of the Syk structure. As shown in the Figure S3, MacPyMol representation of the unphosphorylated Syk (PDB 4FL2 Syk[1]) depicted the three Cys residues (101, 593 and 597; dark pink) surrounding by patches of bulky aliphatic groups (Val, Leu and Ile; red) and aromatic (Phe, Tyr and Trp; blue) amino acid residues.

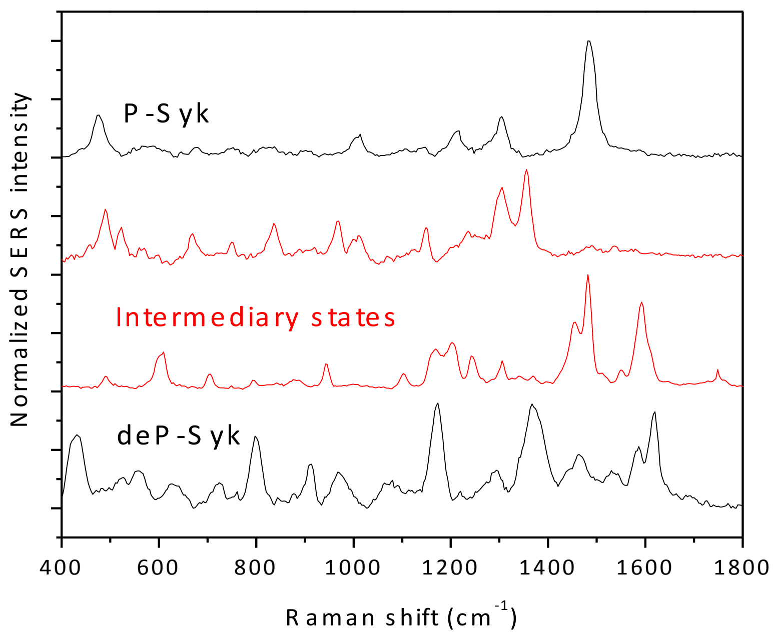

Figure S7. Comparison between normalized SERS spectra of deP-Syk, two partially phosphorylated Syk and P-Syk forms. Two transitional phosphorylated Syk samples (indicated as intermediary states; red) were obtained from the deP-Syk form incubated with ATP for various times.

Table S1. SERS peak assignments of KD-Syk, unP-Syk, deP-Syk and P-Syk based on previous reports[5-12].

KD-Syk	unP-Syk	deP-Syk	P-Syk	Assignment
424	426	427		υ(S-S)
			472	
510, 555	518, 549	525, 558		υ(S-S)
624	618	627		υ(C-S)
648	641	655		Tyr
715	714	724		υ(C-S)
745	749	760		Trp or His
800, 844	791	798		Tyr
		877		Trp
	908	910		υ(C-C)
	940			$N-C_\alpha-C$
	960	968		Trp
996	995	993		Phe (ring stretching)
			1014	Phe or Trp
1073	1063	1062		Non aromatic υ(C-N) et υ(C-C)
	1167	1173		Tyr, Phe
		1219	1214	Phe
1236				Tyr
	1293	1293		δ $(CC_\alpha H)$
1339, 1377, 1397	1336, 1354, 1381	1340, 1366, 1387	1303	Non aromatic δ(CH_2), or Trp (inodole ring)
1449	1435	1440		δ (CH_2)
	1474	1460	1483	δ(CH_2)
1531	1527	1530		Trp
1578	1580	1584		Trp (ring stretching)
1613	1612	1619		Tyr, Phe, Trp (ring stretching)

References

1 Grädler, U. *et al.* Structural and Biophysical Characterization of the Syk Activation Switch. *Journal of molecular biology* 425, 309-333, doi: 10.1016/j.jmb.2012.11.007 (2013).

2 Prisco, U. *et al.* Residue-specific immobilization of protein molecules by size-selected clusters. *Journal of the Royal Society, Interface / the Royal Society* 2, 169-175, doi:10.1098/rsif.2005.0032 (2005).

3 http://jmol.sourceforge.net/index.en.html.

4 Fraczkiewicz, R. & Braun, W. Exact and efficient analytical calculation of the accessible surface areas and their gradients for macromolecules. *Journal of Computational Chemistry* 19, 319–333 doi:10.1002/(SICI)1096-987X(199802)19:3<319::AID-JCC6>3.0.CO;2-W (1998).

5 Siddhanta, S., Karthigeyan, D., Kundu, P. P., Kundub, T. K. & Narayana, C. Surface enhanced Raman spectroscopy of Aurora kinases: direct, ultrasensitive detection of autophosphorylation. *RSC Advances* 3, 4221-4230, doi:10.1039/C3RA22676J (2013).

6 Hu, J., Sheng, R. S., Xu, Z. S. & Zeng, Y. Surface enhanced Raman spectroscopy of lysozyme. *Spectrochimica Acta Part A: Molecular and Biomolecular Spectroscopy* 51, 1087-1096, doi:10.1016/0584-8539(94)00225-Z (1995).

7 Das, G. *et al.* Principal component analysis based methodology to distinguish protein SERS spectra. *Journal of Molecular Structure* 993, 500-505, doi: 10.1016/j.molstruc.2010.12.044 (2011).

8 David, C. *et al.* Raman and IR spectroscopy of manganese superoxide dismutase, a pathology biomarker. *Vibrational Spectroscopy* 62, 50-58, doi: 10.1016/j.vibspec.2012.06.003 (2012).

9 Rygula, A. *et al.* Raman spectroscopy of proteins: a review. *Journal of Raman Spectroscopy* 44, 1061-1076, doi:10.1002/jrs.4335 (2013).

10 Podstawka, E., Ozaki, Y. & Proniewicz, L. M. Adsorption of S-S containing proteins on a colloidal silver surface studied by surface-enhanced Raman spectroscopy. *Appl Spectrosc* 58, 1147-1156, doi:10.1366/0003702042336073 (2004).

11 Brulé, T. *et al.* Sorting of enhanced reference Raman spectra of a single amino acid molecule. *Journal of Physical Chemistry C* 118, 17975-17982 doi: 10.1021/jp504395c (2014).

12 Stewart, S. & Fredericks, P. M. Surface-enhanced Raman spectroscopy of peptides and proteins adsorbed on an electrochemically prepared silver surface. *Spectrochimica Acta Part A: Molecular and Biomolecular Spectroscopy* 55, 1615-1640, doi: 10.1016/S1386-1425(98)00293-5 (1999).
